# Hydrogen Segregation at the Coherent α-Fe/V_4_C_3_ Interface: First-Principles Insights into the Role of Carbon Vacancies

**DOI:** 10.3390/nano16090555

**Published:** 2026-04-30

**Authors:** Linxian Li, Aoxuan Guo, Jiamin Liu, Huifang Lan, Shuai Tang, Zhenyu Liu, Guodong Wang

**Affiliations:** 1 State Key Laboratory of Digital Steel, Northeastern University, Shenyang 110819, China; 2 Research and Development Center, BYD Company Limited, Dapeng District, Shenzhen 518118, China

**Keywords:** density functional theory, vanadium carbide, hydrogen trap, interface

## Abstract

Hydrogen trapping at carbide/matrix interfaces is important for improving the resistance of steels to hydrogen embrittlement. In this work, the segregation behavior of hydrogen at the coherent α-Fe/V_4_C_3_ interface was investigated by first-principles calculations. Representative hydrogen sites were considered systematically, including interstitial sites in the near-interface region, interfacial sites, and carbon-vacancy sites in V_4_C_3_. All of the sites examined are energetically favorable for hydrogen trapping, but the carbon vacancy inside V_4_C_3_ exhibits the strongest trapping tendency. Charge density, Bader charge, and density-of-states analyses indicate that hydrogen at this site gains more electrons and forms stronger interactions with neighboring V atoms, leading to enhanced stability. The behavior of H_2_ at the internal carbon vacancy was also evaluated. After structural relaxation, the H_2_ molecule dissociated into two separate H atoms, indicating that hydrogen is more stably trapped in atomic rather than molecular form. These findings reveal the crucial role of carbon vacancies in regulating hydrogen trapping at the α-Fe/V_4_C_3_ interface and provide atomic-scale insight into the hydrogen trapping mechanism of vanadium carbide precipitates in steels.

## 1. Introduction

Hydrogen embrittlement is a major factor limiting the safe use of high-strength steels [1,2]. Because hydrogen has a small atomic size and high mobility in metallic lattices, it can enter steels during processing or service and subsequently redistribute to microstructural defects such as grain boundaries, dislocations, vacancies, precipitates, and phase boundaries [3,4,5]. The redistribution of hydrogen in the material will significantly change the local bonding state and stress distribution, thus promoting crack initiation and propagation. With the wide application of advanced steel materials in the fields of energy equipment, transportation and engineering structure [6,7,8,9,10], it remains important to clarify how hydrogen interacts with specific microstructural features that control embrittlement susceptibility.

In microalloyed steels, carbide precipitates are widely regarded as important hydrogen traps. In addition to their strengthening effect [11,12,13], these precipitates can reduce the diffusible hydrogen content by trapping hydrogen atoms, thereby lowering susceptibility to hydrogen embrittlement [14,15,16,17,18]. Among transition metal carbides, vanadium carbide (VC) is of particular interest because of its thermal stability and its widespread occurrence in vanadium microalloyed steels [19,20,21,22,23]. However, the hydrogen trapping ability of VC is not only determined by the carbide phase itself, but also closely related to the local atomic structure and electronic structure at the carbide/matrix interface.

From the perspective of real steel microstructures, the α-Fe/carbide interface is more relevant than either bulk α-Fe or bulk carbide alone. Relative to the adjoining bulk phases, the interface region has a distinct atomic arrangement, local strain state, and electronic environment, and may therefore provide segregation sites with different energetics from those in either constituent phase [24]. Using first-principles calculations, Ma et al. pointed out that interfacial strain can strengthen hydrogen binding and increase trap depth at matrix/carbide interfaces [25]. Among them, the α-Fe/V_4_C_3_ interface has important research value, because V_4_C_3_ is one of the typical VC phases in steel [26]. Carbon (C) vacancies are also important when considering hydrogen in vanadium carbides. As common intrinsic defects in VC, they modify the local coordination and bonding environment and are therefore expected to affect hydrogen stability, especially near the interface region [27,28,29]. Boot et al. [30] observed significant hydrogen enrichment in VC by secondary ion mass spectrometry, and pointed out that hydrogen was mainly trapped at the C vacancy inside the carbide. Takahashi et al. [31] believed that the C vacancy near the interface may have higher stability. Therefore, it is of great significance to systematically study the hydrogen behavior at the α-Fe/V_4_C_3_ interface, especially at the defect sites containing C vacancies, for an in-depth understanding of the hydrogen trapping mechanism of VC precipitates.

Despite extensive work on hydrogen trapping in steels [32,33], the atomic-scale behavior of hydrogen at the α-Fe/V_4_C_3_ interface remains insufficiently resolved. In particular, the relative stability of hydrogen at near-interface interstitial sites, interfacial sites, and vacancy-containing sites in V_4_C_3_ has not yet been clarified systematically. This limits a mechanistic understanding of how the interface environment and carbon vacancies jointly influence hydrogen trapping.

In this work, first-principles calculations were performed to investigate hydrogen segregation at the coherent α-Fe/V_4_C_3_ interface. Hydrogen stability was examined at representative sites in the near-interface region, directly at the interface, and at C vacancy sites within V_4_C_3_. By combining energetic analysis with electronic structure characterization, this study distinguishes the respective effects of the interface environment and C vacancies on hydrogen trapping and clarifies the dominant trapping site among the configurations considered.

## 2. Computational Methods

Density Functional Theory (DFT) calculations were performed using the Vienna Ab initio Simulation Package (VASP) [34]. The electron–ion interaction was described by the projector augmented-wave (PAW) method [35], and the exchange-correlation functional was treated within the generalized gradient approximation (GGA) using the Perdew–Burke–Ernzerhof (PBE) scheme [36]. A coherent α-Fe/V_4_C_3_ interface was established based on the Baker–Nutting orientation relationship ((001)_Fe_//(001)$V_{4}C_{3}$, [100]_Fe_//[110]$V_{4}C_{3}$ [37,38]). The model consisted of nine α-Fe layers and nine V_4_C_3_ layers. Since previous studies have demonstrated that the Fe-on-C stacking mode yields the lowest interface energy [39,40,41], this configuration was exclusively considered. A cutoff energy of 400 eV was used for the plane-wave basis set. The computational interface model corresponded to a 3 × 3 in-plane supercell obtained by extending the displayed interface model along both interfacial x and y directions. The total energy and force convergence criteria were set to 1 × 10^−5^ eV/cell and 0.01 eV/Å, respectively. Monkhorst–Pack [42] k-point meshes of 3 × 3 × 1 were used for the interface models. All calculations were performed in a spin-polarized manner, and the α-Fe/V_4_C_3_ interface system was treated in the ferromagnetic state. Structural visualization was performed using VESTA [43].

To characterize the energetics associated with hydrogen trapping, the segregation energy of an interstitial H atom at the α-Fe/V_4_C_3_ interface is defined as follows [44]:

(1) $${∆E}_{\mathrm{seg}}\;=\;{(E}_{\mathrm{tot}}\left(H\right)-E_{\mathrm{tot}}\left(0\right)){\;-\;(E}_{\mathrm{tot}}^{{\mathrm{Fe}}_{\mathrm{bulk}}}\left(H\right)\;-\;E_{\mathrm{tot}}^{{\mathrm{Fe}}_{\mathrm{bulk}}}\left(0\right))$$

where $E_{\mathrm{tot}}\left(H\right)$ and $E_{\mathrm{tot}}\left(0\right)$ are the total energies of the α-Fe/V_4_C_3_ interface with and without a dissolved H atom, respectively. $E_{\mathrm{tot}}^{{\mathrm{Fe}}_{\mathrm{bulk}}}\left(H\right)$ and $E_{\mathrm{tot}}^{{\mathrm{Fe}}_{\mathrm{bulk}}}\left(0\right)$ are the total energies of the Fe supercell with and without a dissolved H atom, respectively. The most stable site for hydrogen in Fe is the tetrahedral interstice [45]. Negative segregation indicates that hydrogen tends to accumulate at that site.

The strain energy of a H atom is defined as the increase in energy caused by lattice distortion when the system traps H atom:

(2) $$E_{\mathrm{strain}}H\;=\;E_{\mathrm{unrelaxed}}\;-\;E_{\mathrm{relaxed}}$$

where $E_{\mathrm{unrelaxed}}$ is the total energy of the system obtained after removing the trapped H while keeping the surrounding structure fixed, and $E_{\mathrm{relaxed}}$ is the total energy of the system after removing the H and then fully relaxing the remaining structure.

## 3. Results and Discussion

### 3.1. Hydrogen Segregation at α-Fe/V_4_C_3_ Interfaces

**Figure 1:**
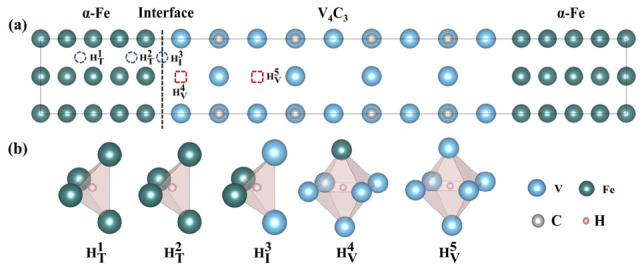
Schematic diagram of the interstitial positions of various H atoms on the interface of α-Fe/V_4_C_3_. (**a**) Overview; (**b**) different hydrogen trapping sites.

To identify favorable hydrogen-trapping sites at the coherent α-Fe/V_4_C_3_ interface, five representative configurations were considered, as shown in Figure 1. $H_{T}^{1}$ denotes the tetrahedral interstitial site inside α-Fe, $H_{T}^{2}$ denotes the tetrahedral interstitial site in the Fe sub-interface layer, $H_{I}^{3}$ denotes the tetrahedral interstitial site in the interface layer, $H_{V}^{4}$ denotes the C vacancy site in the interface layer, and $H_{V}^{5}$ denotes the C vacancy site inside V_4_C_3_.

**Figure 2:**
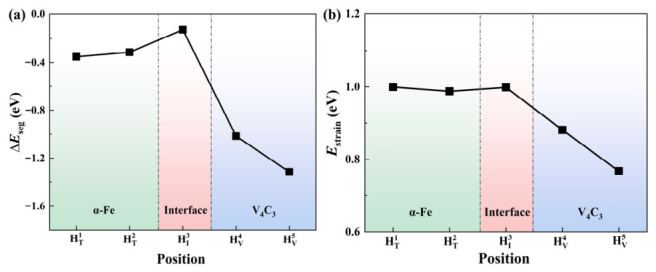
The segregation energy and strain energy of hydrogen at different sites. (**a**) Segregation energy; (**b**) strain energy.

The calculated segregation energies for these sites are summarized in Figure 2a. All five interface structures have negative segregation energies, indicating that each site considered is energetically favorable for hydrogen trapping relative to the chosen reference state. The trapping tendency follows the order $H_{V}^{5}$ > $H_{V}^{4}$ > $H_{T}^{1}$ > $H_{T}^{2}$ > $H_{I}^{3}$, where a more negative segregation energy corresponds to a stronger trapping tendency. Among the sites examined, the carbon vacancy inside V_4_C_3_ ($H_{V}^{5}$) is the most favorable, whereas the interfacial interstitial site ($H_{I}^{3}$) is the least favorable.

To evaluate the structural response to hydrogen incorporation, the strain energies of the five configurations were calculated, as shown in Figure 2b. All strain energies are below 1 eV, which indicates that the incorporation of a single H atom causes only limited lattice distortion in the modeled interface systems. The strain energy follows the order $H_{I}^{3}$ > $H_{T}^{1}$ > $H_{T}^{2}$ > $H_{V}^{4}$ > $H_{V}^{5}$. The $H_{V}^{5}$ configuration has the smallest strain energy (0.77 eV), indicating that the internal carbon vacancy accommodates H with the least local distortion. This is consistent with the segregation-energy results and supports the view that the vacancy inside V_4_C_3_ provides the most favorable trapping environment among the sites considered.

It is worth noting that the deepest trap ($H_{V}^{5}$) has the lowest strain energy. From the thermodynamic point of view, the highly negative segregation energy is a strong driving force for hydrogen capture, and the strain energy plays an inherent elastic penalty role. The interstitial volume of interstitial sites ($H_{T}^{1}$, $H_{T}^{2}$ and $H_{I}^{3}$) is limited, which means that the insertion of hydrogen atoms will naturally cause significant local atomic repulsion and bring higher strain penalty. In contrast, the pre-existing carbon vacancies ($H_{V}^{4}$ and $H_{V}^{5}$) provide relatively large interstitial space, allowing hydrogen atoms to be accommodated with minimal lattice distortion. Since the strain energy at all sites is positive, the segregation energy is mainly dominated by strong electron interaction rather than strain energy.

**Figure 3:**
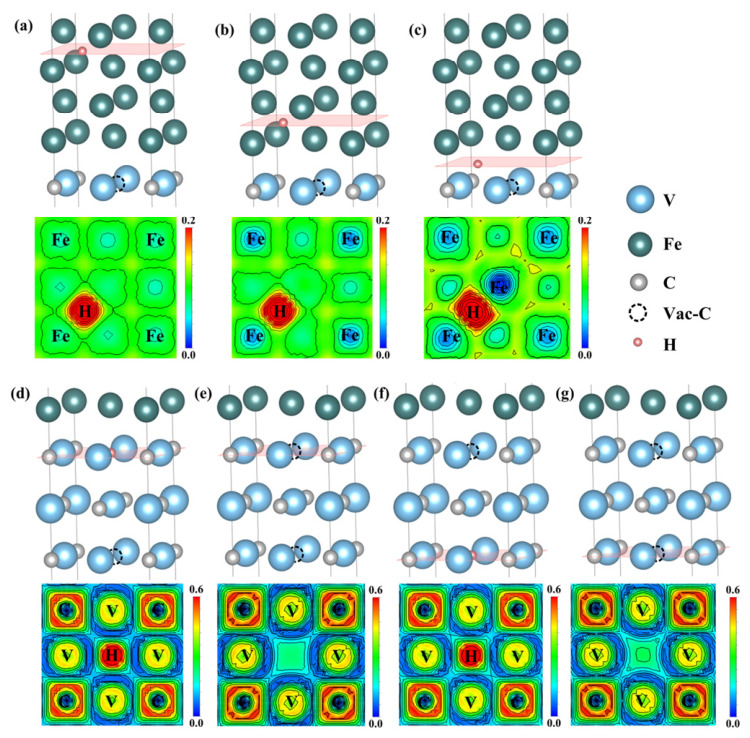
Electronic localization function of (100) crystal plane containing H. (**a**) $H_{T}^{1}$, (**b**) $H_{T}^{2}$, (**c**) $H_{I}^{3}$, (**d**) $H_{V}^{4}$, (**e**) V4, (**f**) $H_{V}^{5}$, (**g**) V5. (Note: Different color scales are used to visualize the distinct bonding characteristics across the interface).

To examine the electronic redistribution associated with hydrogen incorporation, the electron localization function and the corresponding two-dimensional charge-density maps were analyzed for representative planes containing H, as shown in Figure 3. In all configurations, the region around H exhibits strong charge localization. The Fe side is characterized by relatively lower charge density, whereas the V_4_C_3_ side, particularly near C atoms, shows stronger charge accumulation. For the Fe-side sites in Figure 3a–c, the local charge density increases as the H site approaches the interface, indicating that the interfacial region differs electronically from bulk-like α-Fe.

The vacancy-containing configurations show a clearer distinction. Figure 3d,e compare the interfacial carbon-vacancy site before and after H incorporation, while Figure 3f,g show the corresponding results for the carbon vacancy inside V_4_C_3_. In both cases, H incorporation is accompanied by charge redistribution around the neighboring V and C atoms. The internal vacancy in V_4_C_3_ exhibits a higher local charge density than the interfacial vacancy before H incorporation. This suggests that the internal vacancy provides a more electron-rich local environment, which is consistent with its stronger hydrogen-trapping tendency.

The electron localization function indicates that H is more likely to segregate to the C vacancy inside V_4_C_3_ than to the C vacancy at the α-Fe/V_4_C_3_ interface. To quantitatively analyze the charge transfer of H at the interfacial C vacancy ($H_{V}^{4}$) and the internal C vacancy in V_4_C_3_ ($H_{V}^{5}$), the Bader charge transfer of the neighboring atoms on the (100) plane containing H was calculated, as listed in Table 1. As shown in the table, in the H-free α-Fe/V_4_C_3_ system each neighboring C atom around the interfacial C vacancy gains 1.44 electrons and each V atom loses 0.94 electrons. After H is introduced into the $H_{V}^{4}$, each C atom gains 1.46 electrons and each V atom loses 0.98 electrons. The increased electron gain of C and increased electron loss of V indicate that the 0.63 electrons gained by H are mainly supplied by the V atoms. For the H-free α-Fe/V_4_C_3_ system at the $H_{V}^{5}$ inside V_4_C_3_, each neighboring C atom gains 1.54 electrons and each V atom loses 1.02 electrons. After H is introduced, the electron gain of C decreases whereas the electron loss of V increases, providing a larger amount of charge to H. After H is introduced, H gains 0.69 electrons. Thus, H acquires more electronic charge at the internal carbon vacancy than at the interfacial vacancy. This result is consistent with the stronger segregation tendency of $H_{V}^{5}$.

In addition, it can be found that the transition of charge localization behavior at the α-Fe/V_4_C_3_ interface is crucial for hydrogen trapping. As shown in the ELF diagram and confirmed by Bader charge analysis, the electron density in the α-Fe region is mainly non-localized, which is the characteristic of traditional metal bonding. However, as the hydrogen capture sites cross the interface into the V_4_C_3_ layer, the bonding properties change significantly. The charge is highly localized around the hydrogen atom, especially at the C vacancy ($H_{V}^{5}$) inside the V_4_C_3_. This obvious transition (from the widely delocalized electron sea in the Fe matrix to the highly localized and directional charge trapping in the V_4_C_3_ region) fundamentally explains why the hydrogen atoms in the V_4_C_3_ region can trap significantly more localized charges from the surrounding electron-rich V atoms, while the hydrogen atoms in the bulk Fe region cannot do this.

**Figure 4:**
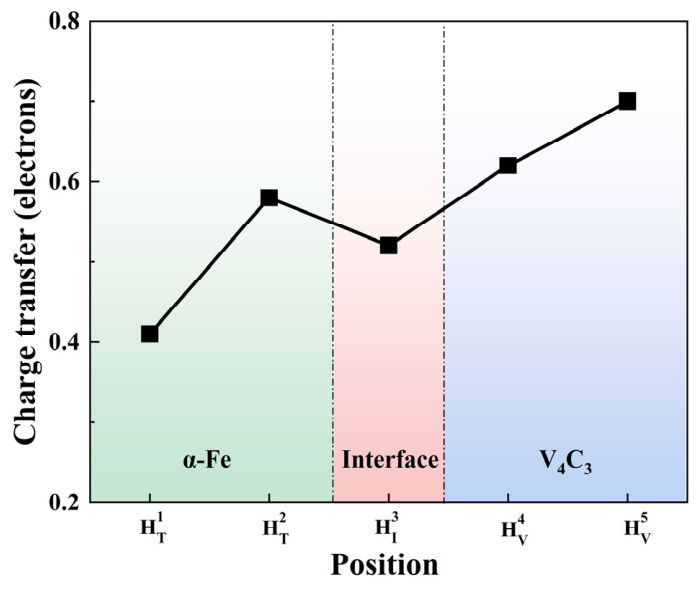
Bader charge transfer of H at different trap positions.

To compare the charge transfer of H at the five defect sites, the Bader charge transfer of H at different defect positions is shown in Figure 4. H atoms at different defect positions gain electrons from the surrounding atoms after structural optimization. The amount of charge gained by H follows the order $H_{V}^{5}$ > $H_{V}^{4}$ > $H_{T}^{2}$ > $H_{I}^{3}$ > $H_{T}^{1}$. In other words, H gains the largest number of electrons at the C-vacancy site in V_4_C_3_. These charge-distribution results indicate that the C vacancy on the V_4_C_3_ side not only contains pre-existing high charge density, but also allows the surrounding V to provide more electrons to H.

**Figure 5:**
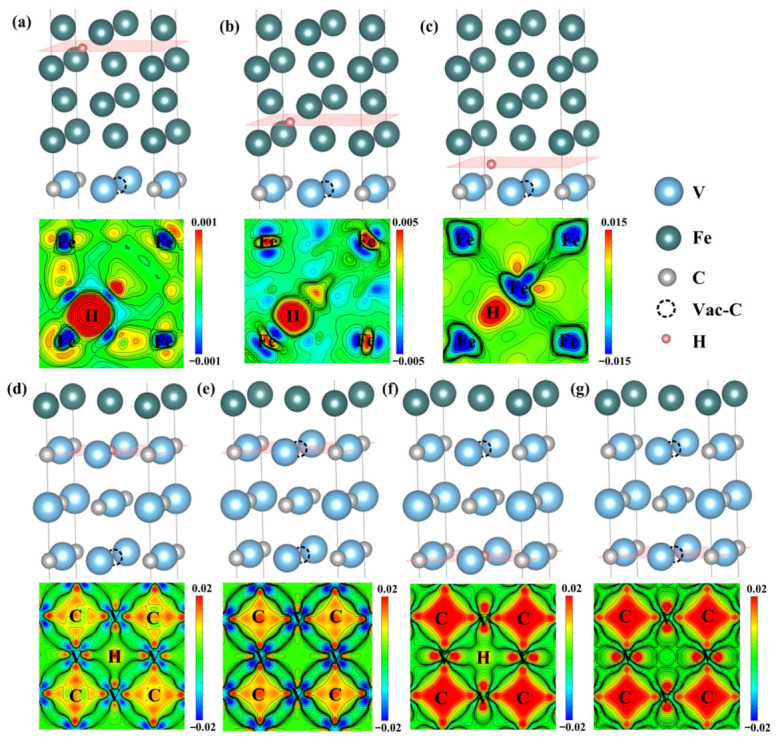
Difference charge density of different defect positions on the (100) crystal plane at the α-Fe/V_4_C_3_ interface. (**a**) $H_{T}^{1}$, (**b**) $H_{T}^{2}$, (**c**) $H_{I}^{3}$, (**d**) $H_{V}^{4}$, (**e**) V4, (**f**) $H_{V}^{5}$, (**g**) V5. (Note: Different color scales are used in the subpanels to render the vastly varying magnitudes of charge redistribution visible, particularly the weak interactions within the bulk Fe matrix).

To further investigate the electronic-structure characteristics of H at different defect sites at the α-Fe/V_4_C_3_ interface, and to discuss bonding, charge transfer, and bond-polarization direction after H occupies different defect sites, the differential charge-density maps for the (100) plane of the H-containing systems were calculated, as shown in Figure 5. According to the charge-density scale in Figure 5, red regions indicate electron gain and blue regions indicate electron loss. Thus, Fe and V atoms lose electrons, whereas C and H atoms gain electrons. The introduction of H changes the charge distribution of the surrounding atoms and the bonding character.

Figure 5a shows a high degree of electron delocalization around α-Fe, together with many delocalized electrons distributed in the surrounding region, indicating the presence of metallic bonding. Figure 5b,c show charge transfer between Fe and H, indicating ionic-bond characteristics. Figure 5d,f show overlap of the electron clouds between V and C atoms, indicating shared electron pairs and the formation of strongly directional covalent bonds between V and C. A small degree of electron-cloud overlap is also observed between H and V, indicating electron sharing and thus covalent interaction between V and H. Moreover, the electron cloud between V and H is asymmetric, implying that the V-H interaction also has an ionic component. Comparison of Figure 5d,f shows that the differential charge density at the C vacancy inside V_4_C_3_ is higher than that at the interfacial C vacancy, indicating stronger V-H bonding at the internal C vacancy site. Comparison of Figure 5e,g further shows that, before H addition, the differential charge density around the internal C vacancy in V_4_C_3_ is higher than that around the C vacancy near the interface, indicating that the V and C atoms around the internal vacancy form stronger polar covalent bonds.

**Figure 6:**
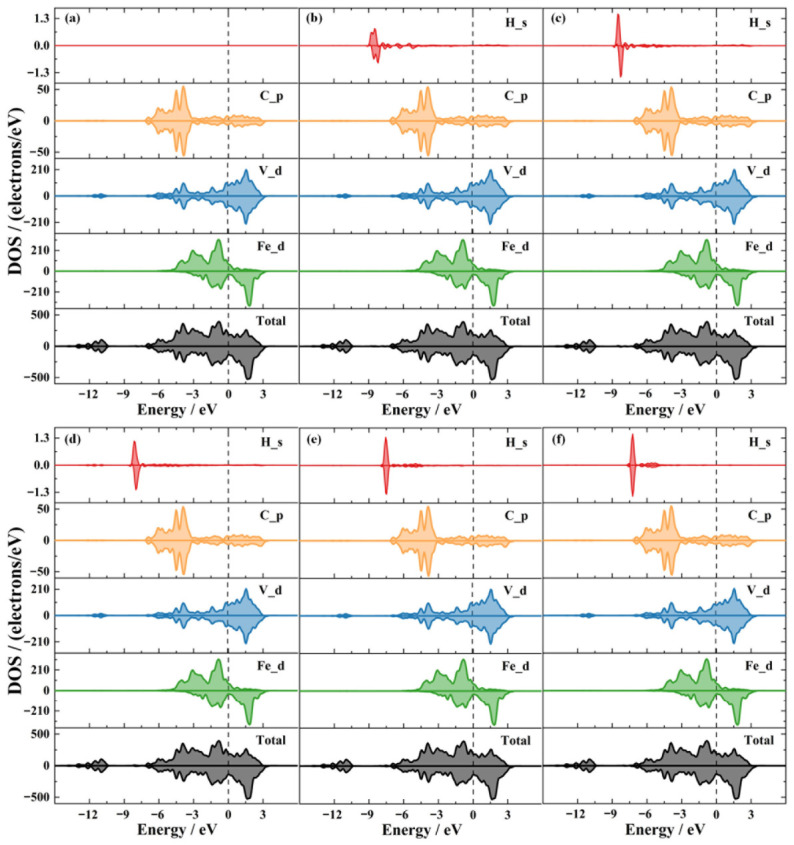
Density of states of different trap positions of H at the interface of α-Fe/V_4_C_3_. (**a**) 0H, (**b**) $H_{T}^{1}$, (**c**) $H_{T}^{2}$, (**d**) $H_{I}^{3}$, (**e**) $H_{V}^{4}$, (**f**) $H_{V}^{5}$. 0H is hydrogen-free interface.

To further analyze the bonding behavior of H at different defect sites at the α-Fe/V_4_C_3_ interface, the total density of states (TDOS) and projected density of states (PDOS) of the systems containing H at the five defect positions were calculated, as shown in Figure 6. The TDOS results show that the spin-up and spin-down total density-of-states curves of the α-Fe/V_4_C_3_ interface are asymmetric, indicating that the interface is ferromagnetic. The nonzero density of states at the Fermi level indicates that the system is metallic and conductive, and the electronic states at the Fermi level are mainly contributed by d orbitals of V. The TDOS curves of the five H-containing interfaces are generally similar, indicating that the introduction of one H atom does not cause a major change in the interfacial density of states.

The DOS curves of the α-Fe/V_4_C_3_ interface can be divided into three regions: a lower valence band, a higher valence band, and a conductive unoccupied band. Specifically, the region from −14.5 eV to −10.5 eV corresponds to the lower valence band and is dominated by d orbitals of V; the region from −9.8 eV to the Fermi level corresponds to the higher valence band and is mainly dominated by d orbitals of V and Fe, and p orbitals of C; and the region from the Fermi level to 4.2 eV corresponds to the conductive unoccupied states and is dominated by d orbitals of V and Fe.

The TDOS can be used to judge the ionic and covalent character of a material. If there is a gap between the lower covalent band and the higher covalent band, the material has ionic character; if the DOS curves of different atoms overlap in the same energy range, the material has a certain degree of covalency. Combined with the PDOS and TDOS shown in Figure 6, the similar peaks of d orbitals of V and Fe, and p orbitals of C in the range from −4.5 eV to −3.89 eV indicate orbital hybridization in this region and thus the formation of relatively strong covalent bonds. The TDOS of the interfaces with and without H also shows a gap between the lower and higher covalent bands, indicating that the interface possesses a certain ionic character. The TDOS curves also show two distinct peaks on either side of the Fermi level, while the density of states between them reaches a non-zero minimum, indicating a pseudo-energy gap. The value of this pseudo-energy gap is determined by calculating the energy distance spanning these two prominent peaks. The larger the pseudo-energy gap, the stronger orbital hybridization and a reduced metallic character. As shown in Figure 6, each system has peaks on both sides of the Fermi level and a nonzero spacing between them, indicating that both the α-Fe/V_4_C_3_ interface and the H-containing defect structures possess a finite pseudo-energy gap and therefore a certain degree of covalent character.

**Figure 7:**
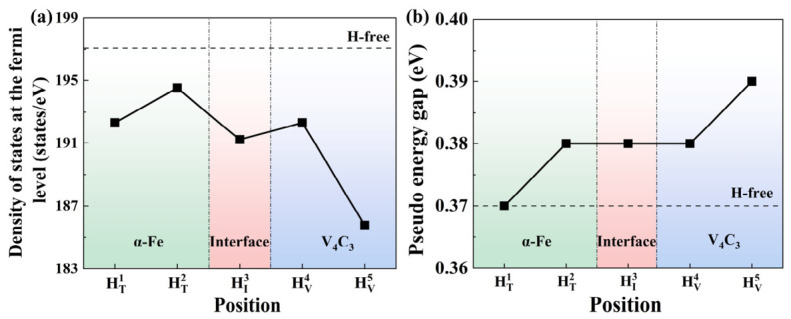
Density of states at fermi level and pseudo-energy gap at different defect positions of α-Fe/V_4_C_3_ interface. (**a**) Density of states at fermi level, (**b**) pseudo-energy gap.

As observed in Figure 6, the TDOS curves of the five H-containing interfaces appear visually similar at a macroscopic level. This global similarity is physically expected, as the introduction of a single H atom into a relatively large supercell acts only as a minor perturbation and does not drastically alter the overall interfacial electronic structure. However, the subtle quantitative differences that govern the specific thermodynamic stability of each trapping site are hidden within these profiles. According to fundamental electronic structure principles, the density of states at the Fermi level reflects the stability of the interface. Physically, a lower DOS at the Fermi level implies that the energetically favorable bonding states are maximally occupied, whereas the higher-energy anti-bonding states remain largely empty. This electronic configuration effectively minimizes the total electronic energy of the system, thereby enhancing its overall thermodynamic and structural stability. Consequently, a lower value at the Fermi level indicates a more stable system. Therefore, to compare the stability of the five configurations, the specific DOS values at the Fermi level extracted from the TDOS in Figure 6 are shown in Figure 7a. The DOS values of all H-containing systems are lower than that of the H-free system, indicating that the incorporation of H lowers the energy and improves the stability of the interface. The DOS values at the Fermi level follow the order 0H > $H_{T}^{1}$ > $H_{T}^{2}$ > $H_{I}^{3}$ > $H_{V}^{4}$ > $H_{V}^{5}$. Therefore, the $H_{V}^{5}$ configuration, where H is trapped at the C vacancy inside V_4_C_3_, has the lowest DOS at the Fermi level and the best structural stability, consistent with the segregation energy.

Figure 7b shows the pseudo-energy gap of the H-free interface and the H-containing interfaces at different defect sites. The interface with H at the tetrahedral interstitial site inside α-Fe ($H_{T}^{1}$) has the narrowest pseudo-energy gap, confirming that the Fe rich region remains dominated by metallic bonding. The pseudo-energy gaps of the interfaces with H at the tetrahedral interstitial site in the Fe sub-interface layer and at the interfacial layer are nearly the same as that of the H-free interface, indicating that H at these two defect sites has little effect on the overall hybridization. In contrast, the interface with H at the C vacancy inside V_4_C_3_ ($H_{V}^{5}$) has the widest pseudo-energy gap. This more pronounced pseudo-gap feature demonstrates that hydrogen trapped at C vacancy sites in the V_4_C_3_ region experiences stronger interactions with neighboring V atoms. This is consistent with enhanced orbital hybridization, a reduced metallic character, and ultimately, greater structural stability at the internal vacancy site.

**Figure 8:**
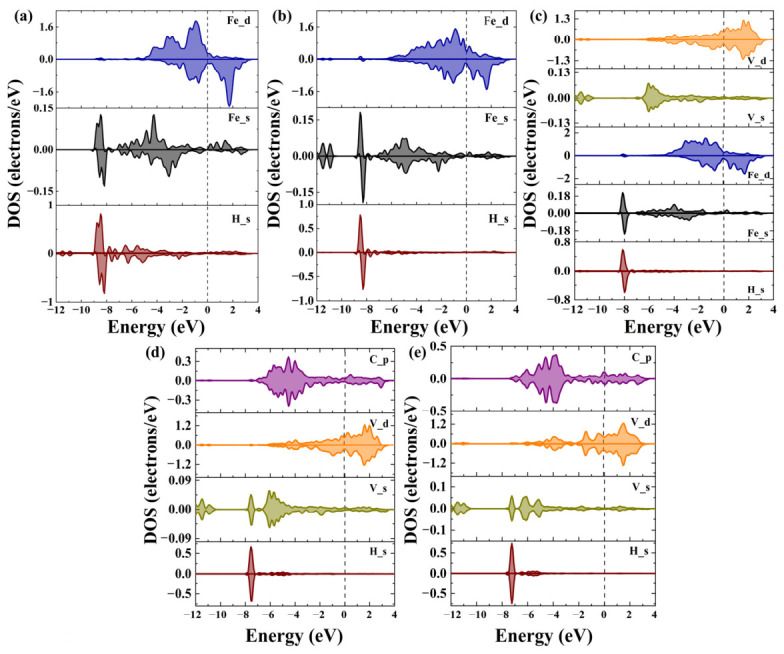
The density of states of H atoms and their nearest-neighbour atoms at different positions. (**a**) $H_{T}^{1}$, (**b**) $H_{T}^{2}$, (**c**) $H_{I}^{3}$, (**d**) $H_{V}^{4}$, (**e**) $H_{V}^{5}$.

To further clarify the interaction between H and the other atoms in the system, the orbital hybridization between H and its neighboring atoms was analyzed, as shown in Figure 8. Figure 8a,b show that when H occupies the tetrahedral interstitial site in Fe, the s orbital of H interacts to different extents with both the s and d orbitals of Fe. The main hybridization occurs in the range from −9 eV to −7.5 eV, and the hybridization between the s orbital of H and the s orbital of Fe is stronger than that between the s orbital of H and the d orbital of Fe. Figure 8c shows that when H occupies the interface layer, the interaction between H and Fe is stronger than that between H and V. In the energy range from −8.5 eV to −7.5 eV, the interaction is mainly between the s orbitals of H and Fe, while the interaction between the s orbital of H and the d orbital of V is slightly stronger than that between the s orbitals of H and V.

Figure 8d shows that when H occupies the C vacancy in V_4_C_3_ near the interface, the main interaction range of the H electronic states is from −7.8 eV to −7.2 eV. The s orbital of H mainly interacts with the neighboring s orbital and d orbital of V, with the strongest interaction occurring between the s orbital of H and the d orbital of V. It is also found that the interaction between H and V when H occupies the interfacial C vacancy site is weaker than that when H occupies the internal C vacancy in V_4_C_3_. This explains why H at the interfacial site moves toward the Fe side after atomic relaxation. When H occupies the C vacancy inside V_4_C_3_, the density of states of the s orbital of H is higher, indicating stronger interaction between H and its neighboring atoms. In summary, at the α-Fe/V_4_C_3_ interface, the main reason why H tends to segregate to the C vacancy region on the V_4_C_3_ side is the strong interaction between the s orbital of H and the d orbital of V. This further confirms that H preferentially occupies interstitial or defect sites with higher charge density.

To understand whether magnetic polarization influences the trapping stability, the local magnetic moments of the trapped H atoms were systematically analyzed. The calculated magnetic moments of H are approximately −0.025, −0.022, −0.009, −0.002, and 0 μB for the $H_{T}^{1}$, $H_{T}^{2}$, $H_{I}^{3}$, $H_{V}^{4}$, and $H_{V}^{5}$ configurations, respectively. These exceptionally small values indicate that hydrogen carries only a negligible induced magnetic moment across all considered configurations. Furthermore, the magnitude of the H magnetic moment progressively decreases as the trapping site moves from the Fe-rich region toward the V_4_C_3_ side, becoming essentially zero at the internal carbon-vacancy site $H_{V}^{5}$. This negligible magnetic response explicitly demonstrates that the deep trapping behavior and site preference of hydrogen in the α-Fe/V_4_C_3_ system are primarily governed by strong local electronic interactions and orbital hybridization, rather than by the magnetic polarization of the hydrogen atom itself.

### 3.2. Stability of H_2_ Molecule at the $H_{V}^{5}$ Site

The preceding results identify the carbon vacancy inside V_4_C_3_ ($H_{V}^{5}$) as the most favorable trapping site for a single H atom at the α-Fe/V_4_C_3_ interface. A further question is whether hydrogen at this site remains in atomic form or can be stabilized as an H_2_ molecule. To examine this issue, an H_2_ molecule with an initial H-H bond length of 0.75 Å was placed at the center of the internal carbon vacancy while maintaining the symmetry of the interface structure (α-Fe/V_4_C_3_-H_2_), and structural relaxation was then performed. After relaxation, the segregation energy of α-Fe/V_4_C_3_-H_2_ system is −0.19 eV. Although the negative segregation energy indicates that this site can trap two H atoms, the optimized structure shows a pronounced displacement of H, and the H-H bond length is no longer preserved. The segregation energy is much higher than that of $H_{V}^{5}$ (−1.31 eV), which shows that the internal C vacancy preferentially stabilizes atomic H rather than molecular H_2_.

**Figure 9:**
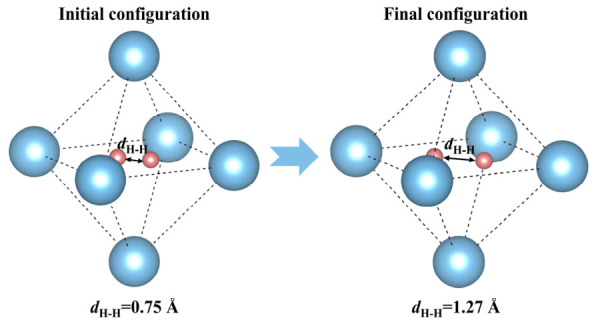
The schematic diagram of the change in H-H bond length at C vacancy in V_4_C_3_ at α-Fe/V_4_C_3_ interface.

Figure 9 shows the change in H-H bond length before and after relaxation. After relaxation, the relative positions of the two H atoms change, the H-H bond breaks, and the two H atoms move toward the nearest neighboring V atoms and finally stabilize around the vacancy. The H-H distance increases from 0.75 Å to 1.27 Å, by 69%, and no longer corresponds to the bond length of an H_2_ molecule.

**Figure 10:**
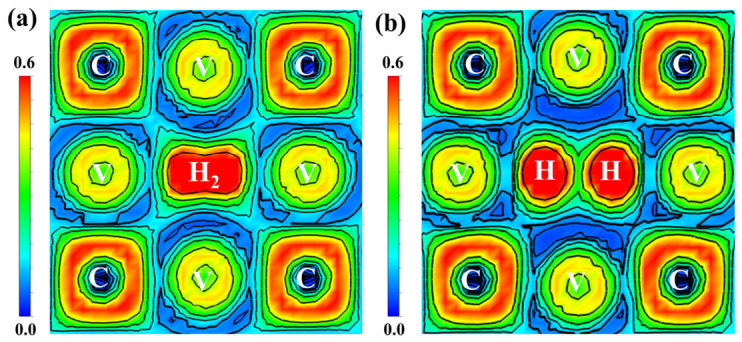
The local charge density of (100) crystal plane before and after H_2_ relaxation at α-Fe/V_4_C_3_ interface. (**a**) Before relaxation, (**b**) after relaxation.

To explore why H does not exist as an H_2_ molecule at the vacancy, the electronic localization function and differential charge density of the α-Fe/V_4_C_3_-H_2_ supercell were calculated. Figure 10 shows the local charge-density maps of the α-Fe/V_4_C_3_-H_2_ interface on the (100) plane before and after atomic relaxation. Before relaxation, electron-cloud overlap appears between the two H atoms at the vacancy, indicating that the two H atoms share electrons and form an H-H covalent bond. After relaxation, a charge-depletion region appears between the two H atoms, indicating repulsion interaction between them. This demonstrates the dissociation of H_2_.

**Figure 11:**
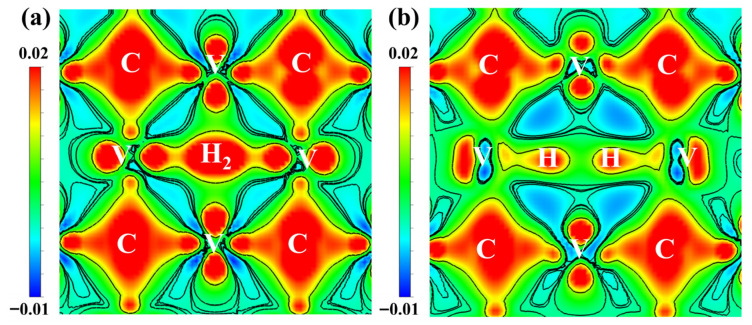
The difference charge density of (100) crystal plane before and after H_2_ relaxation at α-Fe/V_4_C_3_ interface. (**a**) Before relaxation, (**b**) after relaxation.

To further discuss the bonding between the H_2_ species at the vacancy and the neighboring atoms in α-Fe/V_4_C_3_-H_2_, the differential charge density before and after atomic relaxation was calculated, as shown in Figure 11. When an H_2_ molecule is introduced into the C vacancy at the α-Fe/V_4_C_3_ interface and then relaxed, the charge around the vacancy is redistributed. Charge depletion regions appear around the V atoms, and the charge distribution becomes directional. The charge of the two surrounding V atoms is transferred toward the H atoms, indicating a certain ionic character. A charge-depletion region appears between the two H atoms, the H-H bond breaks, and the H atoms move toward the neighboring V atoms, receiving electrons from them and forming strong polar covalent V-H bonds. The charge distribution around the C atoms also becomes directional because the interaction between H and V weakens the V-C covalent bond. Taken together, the structural, energetic, and electronic results indicate that H preferentially exists around the internal C vacancy in V_4_C_3_ at the α-Fe/V_4_C_3_ interface in atomic form rather than as an H_2_ molecule.

## 4. Conclusions

This study used first-principles calculations to examine hydrogen segregation at the coherent α-Fe/V_4_C_3_ interface. Representative hydrogen sites in the Fe region, at the interface, and at carbon-vacancy sites in V_4_C_3_ were compared in terms of segregation energy, strain energy, and electronic structure. The possibility of accommodating H_2_ at the most favorable vacancy site was also evaluated. The main conclusions are as follows:

(1) The segregation energy results indicate that the segregation energy and strain energy of H at the C vacancy site inside V_4_C_3_ is the lowest. The local charge density map and Bader charge transfer indicate a high charge density reserved at this position, revealing that H atoms tend to segregate in areas with high charge density. The difference charge density map shows that the introduction of H atoms into the C vacancy results in the highest electron gain, and the H atom forms a polar covalent bond with the surrounding V atoms. According to the DOS, the electronic density of states at the fermi level of the H atom in the C vacancy within V_4_C_3_ is the lowest, with the best stability, the widest pseudo-energy gap, and the strongest covalent character, indicating that H atoms tend to segregate in the C vacancy within V_4_C_3_ rather than at the interface.

(2) The segregation energy of H_2_ molecules at the C vacancy at the α-Fe/V_4_C_3_ interface is much higher than that of H atom. H_2_ cannot stably exist in the C vacancy. Electronic structure analysis reveals that after atomic relaxation, the H-H bond breaks, and a charge depletion region appears between the two H atoms at the vacancy. The charges around H move towards the neighboring V atoms, forming a strong V-H polar covalent bond.

## Figures and Tables

**Table 1:** Bader charge transfer at the $H_{V}^{4}$ and $H_{V}^{5}$ sites (electrons).

Site	Neighboring Atom	Charge Gain/Loss (Without H)	Charge Gain/Loss (with H)	Site	Neighboring Atom	Charge Gain/Loss (Without H)	Charge Gain/Loss (with H)
$H_{V}^{4}$	C53	1.44	1.46	$H_{V}^{5}$	C54	1.54	1.51
	C66	1.44	1.46		C67	1.54	1.51
	C92	1.44	1.46		C93	1.54	1.51
	C105	1.44	1.46		C106	1.54	1.51
	V81	−0.94	−0.98		V82	−1.02	−1.04
	V86	−0.94	−0.98		V87	−1.02	−1.04
	V99	−0.94	−0.98		V100	−1.02	−1.04
	V140	−0.94	−0.98		V141	−1.02	−1.04
	H1	-	0.63		H1	-	0.69

## Data Availability

The original contributions presented in the study are included in the article; further inquiries can be directed to the corresponding author.
